# A Method for Positive and Negative Selection of *Plasmodium falciparum* Platelet-Mediated Clumping Parasites and Investigation of the Role of CD36

**DOI:** 10.1371/journal.pone.0055453

**Published:** 2013-02-06

**Authors:** Mònica Arman, Yvonne Adams, Gabriella Lindergard, J. Alexandra Rowe

**Affiliations:** Centre for Immunity, Infection and Evolution, Institute of Immunology and Infection Research, School of Biological Sciences, University of Edinburgh, Edinburgh, United Kingdom; University of Copenhagen, Denmark

## Abstract

Platelet-mediated clumping of *Plasmodium falciparum* infected erythrocytes (IEs) is a frequently observed parasite adhesion phenotype. The importance of clumping in severe malaria and the molecular mechanisms behind this phenomenon are incompletely understood. Three platelet surface molecules have previously been identified as clumping receptors: CD36, globular C1q receptor (gC1qR/HABP1/p32), and P-selectin (CD62P), but the parasite ligands mediating this phenotype are unknown. The aim of this work was to develop a selection method to facilitate investigations of the molecular mechanisms of clumping in selected *P. falciparum* lines. Magnetic beads coated with anti-platelet antibodies were used to positively and negatively select clumping IEs from *P. falciparum* strains IT, HB3, 3D7 and Dd2. Clumping in all four positively selected parasite lines was abolished by antibodies to CD36, but was not affected by antibodies to gC1qR or P-selectin. Clumping positive lines showed significantly higher binding to CD36 than clumping negative lines in flow adhesion assays (strains IT, HB3 and 3D7, p<0.05 for all strains, paired t test) and static assays (strain Dd2, p<0.0001 paired t test). However, clumping negative lines IT, HB3 and 3D7 did show some binding to CD36 under flow conditions, indicating that CD36-binding is not sufficient for clumping. These data show that CD36-dependent clumping positive and negative lines can easily be selected from *P. falciparum* laboratory strains. CD36-binding is necessary but not sufficient for clumping, and the molecular differences between clumping positive and negative parasite lines responsible for the phenotype require further investigation.

## Introduction

Platelet-mediated clumping (abbreviated to “clumping”) of *P. falciparum* infected erythrocytes (IEs) results from *in vitro* binding interactions between mature pigmented-trophozoite IEs and platelets [1], [2]. The clumping phenotype is commonly detected in parasites obtained from malaria patients (clinical isolates) and culture-adapted laboratory strains. In the case of clinical isolates, the clumping phenotype has been associated with severe malaria in some studies [1], [3], [4], [5], but with high parasitaemia (Pt) and not severe disease in another [6]. A detailed characterization of the *in vitro* assay used to assess clumping revealed that experimental conditions such as haematocrit (Ht) and Pt have a profound effect on the outcome of the assay [2]. These conditions were not standardized in many of the early studies on clumping and malaria severity, which are therefore biased due to higher Pt in the severe malaria group. Better controlled assays in which the Pt and Ht of samples from uncomplicated and severe malaria groups were adjusted have been used more recently with samples from Malawi [4] and Mozambique [5], however, the numbers of isolates studied remains small and the association between clumping and clinical severity requires further investigation.

The molecular mechanisms behind IE-platelet interaction are poorly understood. To date, three platelet surface molecules have been identified as receptors for clumping: CD36 [1], [4], gC1qR [7], and P-selectin/CD62P [4]. While CD36-dependent clumping seems to be the most common form, it has been proposed that gC1qR-mediated adhesion could be associated with more severe forms of disease [7]. Nothing is yet known about the parasite ligand(s) involved in binding to platelets. *P. falciparum* IEs can show a wide range of cytoadhesion phenotypes other than clumping, such as rosetting (binding of IEs to uninfected Es), binding to endothelial cell surface molecules such as CD36 and ICAM-1, and binding to chondroitin sulfate proteoglycans on placental syncytiotophoblasts (reviewed in [8]). These cytoadherent properties are known to be mediated by *Plasmodium falciparum* Erythrocyte Membrane Protein One (PfEMP1) variant surface antigens (parasite adhesins exported to the surface of the IE) encoded by the *var* gene family [9]. However, the role of PfEMP1 and other variant surface antigen families in platelet-mediated clumping of IEs has not yet been evaluated. The lack of a selection method for *P. falciparum* clumping has been a limiting factor in studying the molecular mechanisms of parasite-platelet interaction.

The aim of this study was to set up a selection method for *P. falciparum* clumping to facilitate further investigation of the molecular mechanisms underlying this phenotype. Isogenic clumping positive and negative parasite populations were successfully derived for four laboratory strains, and platelet CD36 was confirmed as a major receptor for clumping.

## Materials and Methods

### Ethics Statement

Human blood and serum for parasite culture and platelet purification were collected from volunteer donors after written informed consent and protocols were approved by the Scottish National Blood Transfusion Service Committee for the Governance of Blood and Tissue Samples for Non-Therapeutic Use (Reference no. 04-49).

### *P. falciparum* Cultures

The *P. falciparum* laboratory strains used in this study were IT clone A4, Dd2, HB3, and 3D7. The IT/A4 clone is derived from the IT/FCR3 strain [10]. Parasites were cultured in RPMI 1640 medium (Lonza, catalogue number 12-167F) supplemented with 2 mM glutamine, 25 mM Hepes, 20 mM glucose, 25 µg/ml gentamicin, and either 10% pooled human serum or 5% serum +0.25% Albumax II (Invitrogen) [11], with the pH adjusted to 7.2–7.4 with 1 M NaOH. Cultures were set up at 1% haematocrit with blood group O erythrocytes (donors from the Scottish National Blood Transfusion Service) and incubated at 37°C with 3% CO_2_, 1% O_2_, and 96% N_2_. Cultures were synchronized by sorbitol treatment as previously described [12]. The health and maturity of cultures were monitored by daily examination of thin blood smears stained with 10% Giemsa for 15–20 min.

### Human Platelet-Rich Plasma (PRP)

Whole blood from healthy volunteers was collected into citrate-phosphate-dextrose (CPD) and either used fresh or stored at 4°C and used within seven days [2]. Platelet-mediated clumping selections and pH experiments were performed with platelets obtained from stored blood, while both fresh and stored blood was used as a source of platelets for the antibody inhibition described below. PRP was isolated by centrifugation of whole blood for 10 min at 250 *g* and collecting the upper fraction. Platelet-poor plasma (PPP) was obtained by further centrifugation of the PRP fraction (1500 *g*, 30 min) and collection of the upper fraction. Platelet concentration was measured using a Neubauer haemocytometer. PRP and PPP were prepared freshly for each experiment.

### Platelet-mediated Clumping Assay

Clumping frequency was determined using the *in vitro* assay described by Pain *et al* [1] with some modifications [2]. Parasite cultures (at mature pigmented-trophozoite stage) were stained with 25 µg/ml of ethidium bromide for 5 min at 37°C, and then used to set up mixtures of parasite culture and platelets in a final volume of 300–500 µl in Eppendorf tubes. All assays were prepared in binding medium (BM), which is RPMI 1640 without sodium bicarbonate, containing 2 mM glutamine and 25 mM Hepes (Invitrogen, catalogue number 13018015) supplemented with 20 mM glucose and 25 µg/ml gentamicin. Unless otherwise stated, experimental conditions were pH 7.3, 1% parasitemia (Pt), 10% haematocrit (Ht), and 20% PRP or PPP (final platelet concentration ∼3×10^7^ platelets/ml). The cell suspensions were gently rotated (10 rpm) on a wheel at room temperature, and two wet preparations were made per tube after 1 h incubation, unless otherwise stated. Wet preparations (10 µl of culture suspension on a microscope slide beneath a 22×22 mm coverslip with the edges sealed by nail varnish) were viewed on a fluorescence microscope (400X magnification) and 500 IEs were counted and scored for clumping from each slide. A clump consisted of three or more IEs sticking together. Clumping Frequency (CF) is expressed as the percentage of IEs in clumps out of 500 IEs counted. In some experiments, clumping frequency in PPP was used to control for the possibility of IE aggregation occurring without platelet binding.

#### Selection of *P. falciparum* cultures for the platelet-mediated clumping phenotype

**Figure 1:**
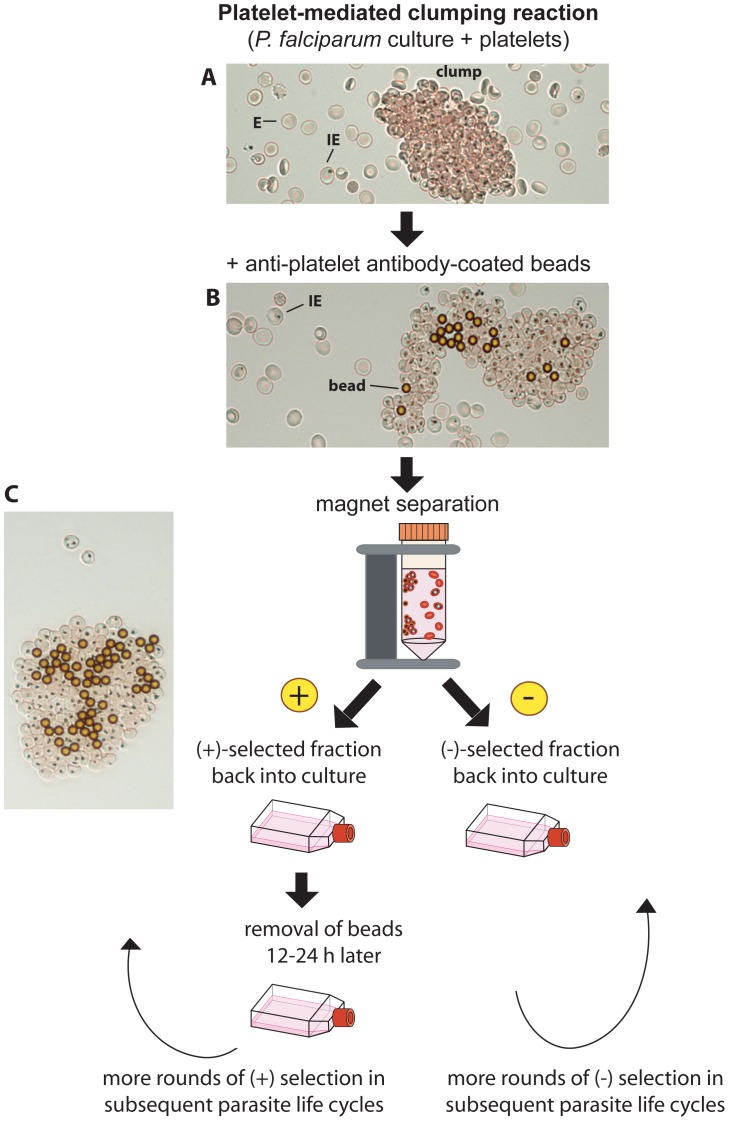
Schematic diagram of positive and negative selection for *P. falciparum* platelet-mediated clumping. (A) A platelet-mediated clumping assay is carried out in sterile conditions by co-incubating an unstained mature pigmented trophozoite-stage *P. falciparum* culture with platelets in suspension. The presence of clumps is confirmed by viewing a wet preparation of parasite culture suspension using bright-field microscopy (400× magnification). Infected erythrocytes (IEs) can be distinguished from erythrocytes (Es) by the presence of pigment (black dots) in IEs that corresponds to the parasite-generated haemozoin. The platelets cannot be seen in these unstained preparations (for visualization of platelets in clumps see references [1], [2]). (B) Magnetic beads coated with an anti-platelet antibody are added to the suspension and become bound to the platelets within the clumps. Binding of beads is confirmed by viewing a wet preparation of parasite culture suspension using bright-field microscopy (400× magnification). (C) A magnet is applied to separate beads and bead-bound clumps (positive fraction, bound to the magnet) from non-clumping IEs and Es (negative fraction, remaining in the supernatant). Each fraction is washed and transferred to a new flask with fresh uninfected Es added as required. The beads can be magnetically separated from the positive fraction the following day after the parasites have invaded fresh E to become non-adherent ring stages. The clumping frequency (CF) of the selected populations can be tested in the next cycle, with further rounds of (+) or (−) selection being performed as required.

A method based on magnetic separation using beads coated with an anti-platelet monoclonal antibody (mAb) was applied. Briefly, after setting-up a clumping assay, antibody-coated beads were added to the suspension. The beads bound to the platelet-IE clumps, which were then separated from the rest of cell suspension by means of a magnet (see Figure 1).

#### Coating beads with anti-platelet antibody

4.5 µm diameter-Dynabeads M-450 Epoxy (Dynal, Invitrogen) were coated with the CD62P (P-selectin) mAb Clone CLB-Trhomb/6 (Immunotech, Beckman Coulter). Coating was performed according to the manufacturer’s instructions; 3 µg of anti-CD62P mAb were used per 25 µl of Dynabead stock (1×10^7^ Dynabeads). Coated beads were kept at 4°C in sterile PBS/0.1% BSA for up to two weeks.

#### Platelet-mediated clumping selections

Starting with an unselected *P. falciparum* culture, a first selection was performed from which two independent populations were obtained, clumping positive (Clump+) and clumping negative (Clump−). The Clump+ fraction, which was composed of clumps (platelets and IEs) and beads, was put back into culture with the addition of fresh uninfected Es. The Clump− fraction, which includes uninfected Es plus non-clumping IEs was also returned to culture. The beads were removed from the Clump+ culture the next day when the parasites were non-adherent ring stages. Up to five repeated rounds of positive or negative selection were carried out in subsequent asexual cycles to obtain highly enriched Clump+ or Clump− populations. Experimental conditions used for the first selection are described below. Subsequent positive selections use the same conditions, whereas subsequent negative selections use the adjusted conditions described below.

#### Experimental conditions for first selection

A clumping assay in sterile conditions without ethidium bromide was performed at 5% Ht, 5% Pt, and 10% PRP, in RPMI 1640 BM pH 7.3 (as described above under clumping assay), and incubated at RT for 30 min at 10 rpm. For example, for 250 µl packed cell volume (pcv) at 5% Pt, a 5 ml suspension was set up in a 15 ml Falcon tube. After the incubation, the Ht was lowered to 2.5% (by adding an equal volume of BM) and antibody-coated beads were added (approximately 50–100 µl of coated beads for 250 µl pcv). Cells were incubated with beads for 30 min. A wet preparation (done without compromising the sterility of the culture) was prepared at this point to check that the beads were bound to the clumps (Figure 1). If too few beads were seen per clump (less than 1 bead per 10–20 IEs), more beads were added and incubation continued for a further 30 mins.

#### Positive selection

A magnet (Ambion, Life Technologies, Catalogue No. 10025) was then applied to isolate the Clump+ fraction. If the Clump− fraction was required, the supernatant was transferred to a fresh tube and the procedure continued as described under “negative selection” below. The Clump+ fraction was washed twice in BM using the same volume as that used during bead incubation, i.e. 10 mls if following the conditions described above. The magnet was used to separate the magnetic bead-bound cells from the supernatant between the washes. Thorough washes are important in order to get rid of as many non-clumping IEs as possible. Finally the Clump+ fraction was collected with complete medium including 1–2% Ht of uninfected Es and put back into culture. The culture was mixed well by vigorous pipetting in order to partially disrupt clumps and facilitate merozoite reinvasion of fresh Es. The pcv of the final culture should be approximately the same (or less) as the starting pcv that was used for the selection. The beads were removed the next day when IEs are at the ring-stage; for this, the culture was transferred to a Falcon tube (15 or 50 ml depending on the culture volume) and the magnet applied. The supernatant fraction was recovered and cultured as usual.

#### Negative selection

After the first selection (see above), experimental conditions used for subsequent rounds of negative selection included higher Ht and Pt values, more PRP, and longer incubations. These conditions favour the formation of giant clumps, which are easy to remove. For example, a clumping assay of 250 µl pcv at 10% Pt, 10% Ht, and 30% PRP (750 µl) was set-up in a 15 ml Falcon tube. The suspension was incubated for 1 h 30 min. The Ht was then lowered to 5% by adding an equal volume of BM, and 50 µl of coated beads were added and incubated for 1 h. Wet preparations were used to monitor the selection; if very few beads were seen per clump, more beads were added (approximately 1 or more beads per 5 clumping IEs gives a good separation). After magnetic separation, the soluble unbound fraction (Clump−) was taken without disturbing the (Clump+) fraction. If clumps (without beads) were still seen in the recovered Clump− fraction, another incubation step with fresh beads was performed. The final clumping- population was washed in BM, and the pcv and Pt measured. A final culture at 1–2% Ht, 1–2% Pt in complete RMPI 1640 was prepared by adding fresh Es, and cultured as usual.

#### Checking the clumping frequency of selected cultures

In the next asexual cycle, or as soon as the selected population reaches 1% Pt, the clumping frequency of the selected culture should be checked as described above. If necessary, new rounds of positive and/or negative selection can be performed.

### Fixed Cell ImmunoFluorescence Assay (IFA) to Determine Knob Positivity of *P. falciparum* Lines Selected for Platelet-mediated Clumping

Thin blood smears of Clump+ and Clump− selected parasite lines were air dried and fixed with cold 90% acetone/10% methanol for 30 mins. Primary incubation was with mAb 89 to the Knob-Associated Histidine Rich Protein (KAHRP, sometimes known as Histine-Rich Protein 1, HRP-1) [13] or mouse IgG2a isotype control (both at 10 µg/ml, dilutions in PBS/1%BSA). The slides were incubated for 1 h at room temp in a humid box, then washed three times for five mins per wash in PBS in a staining jar. Secondary incubation was for 45 mins at room temp with 1/500 dilution of Alexa Fluor 488 highly cross-absorbed goat anti-mouse IgG (Invitrogen) in PBS/1% BSA plus 1 µg/ml 4,6-DiAmidino-2-PhenylIndole (DAPI) to stain parasite nuclei. Slides were then washed as above, air-dried and a coverslip added mounted with a drop of 1.25 mg/ml of DiazaBiCyclo- [2,2,2]Octane (DABCO) in 50% glycerol/50% PBS. The edges of the coverslip were sealed with nail varnish. Slides were viewed using a Leica DM LB2 fluorescence microscope and the percentage of IEs showing fluorescent staining out of 100 IEs counted with a 100X objective was assessed. Counts of 100 IEs were made on four separate areas of the slide and the mean and SE of the four counts was calculated. Images were taken with a Leica DFC300FX digital camera and equivalent exposures were used for isogenic Clump+ and Clump− parasites.

### Inhibition of Platelet-mediated Clumping of *P. falciparum* IEs by Anti-platelet Antibodies

Clumping assays were performed with platelets pre-incubated with antibodies against different human platelet receptors as previously published [1], [4], [7], with minor modifications. Antibodies used in the present study were: isotype control IgMκ clone MM-30 (BioLegend), anti-CD36 mAb clone SMφ (IgM, AbDSerotec), isotype control IgG1κ MG1-45 (Biolegend), anti-CD36 mAb clone FA6-12 (IgG1, Beckman Coulter), anti-CD62P mAb clone AK-6 (IgG1, AbDSerotec), anti-CD31 mAb clone WM59 (IgG1κ, Biolegend), anti-CD41 mAb clone P2 (IgG1κ, Beckman Coulter) and anti-gC1qR mouse polyclonal (IgG, Abcam ab88591). Briefly, PRP was diluted ¼ in BM pH 7.3 that included the corresponding antibody (or a control without antibody) and incubated for 45 min at 37°C. Washed parasite suspensions (stained with ethidium bromide) were then added to the mixture to assess clumping, with the final assay conditions being 10% Ht, 1% Pt, 20% PRP, 1 h incubation. All antibodies were tested at a final concentration of 20 µg/ml apart from anti-CD36 mAb SMφ (also tested at 2 and 0.2 µg/ml) and anti-CD36 mAb FA6-12 (tested at 10, 1, 0.1 and 0.01 µg/ml). Platelet concentration after incubating PRP with or without antibodies was checked by counting in a Neubauer haemocytometer; none of the antibodies used here caused platelet aggregation at the concentrations and experimental conditions used. One experiment each for IT and HB3 were carried out using PRP from fresh rather than stored blood. In this case, blood was collected into CPD without the use of a tourniquet, and kept at room temperature and used within 5 hours of venepuncture. PRP was obtained as described above.

### CD36 Binding Assays under Flow

Microslide capillary tubes pre-treated with 3-Aminopropyl triethoxysilane were coated for 2 h at 37°C with CD36 (recombinant human CD36/SR-B3 Fc Chimera, catalogue number 1955-CD, R&D Systems) at 5 µg/ml, and then blocked overnight at 4°C with PBS/1% BSA. Microslides were then mounted on the stage of an inverted phase-contrast microscope and connected to a flow system. IE suspensions at 3% Pt and 1% Ht in BM pH 7.3 or pH 6.8 were flowed over for a total of 5 min, and then binding buffer flowed over for 5 min at the same rate to remove unbound cells. The flow rate yielded a wall shear stress of 0.05 Pa (calculated based on the formula in [14]), which has been used widely to mimic wall shear stresses in the microvasculature. All assays were performed at 37°C. For each experiment, the numbers of stationary IEs per 20× magnification microscope field were counted by direct microscopic observation in six separate areas on the microslide. In addition to microslide capillary tubes, experiments were also conducted using recombinant human CD36 coated μ-slide I chambers in conjunction with an ibidi perfusion system (ibidi GmbH, München, Germany), to produce the desired wall shear stress of 0.05Pa. The μ-slide I chambers are supplied pre-coated with ibiTreat to facilitate the attachment of soluble proteins. As before, μ-slide I chambers were coated for two hours with 5 µg/ml recombinant human CD36 and blocked over night at 4°C with PBS/1% BSA. The following day, 5 ml of IE suspension was adjusted to 3% Pt and 1% Ht in BM pH 7.3, then added to the perfusion system and allowed to flow over the μ-slide I for 5 minutes at 0.05 Pa, before washing for 5 minutes to remove unbound cells. The numbers of stationary IEs per 20× microscope field (inverted phase-contrast) were counted on six separate areas of the μ-slide. All binding assays (flow and static) were carried out in the absence of platelets, therefore there would not be any clumps present in the culture that might interfere with binding.

### Static Binding Assays

Static binding assays were conducted as previously published [10] with minor modifications, in order to assess the ability of strain Dd2 IEs to adhere to CD36 (25 µg/ml of recombinant human CD36/SR­B3 Fc Chimera, R&D Systems). Briefly, three µl spots of recombinant protein (diluted in PBS) were absorbed on plastic dishes (BD Falcon no. 351007) in a moist atmosphere at 4°C overnight. Control spots contained PBS only. Next day the spots were aspirated and the dish was blocked with PBS/2% BSA for 2 hours at 37°C. A suspension of parasite culture at pigmented-trophozoite stage (1.5 ml) in BM/1% BSA pH 7.0 at 2% Ht and 3% Pt was placed in each dish, and incubated at 37°C for 60 min, resuspending the cells every 12 min. Unbound cells were removed from the dish by carefully washing with BM using a plastic pasteur pipette to gently add and remove wash buffer at the edge of the dish. The adherent cells were fixed with 2% glutaraldehyde, stained with 5% Giemsa for 15 mins, and the number of cells per microscope field (1000× magnification, light microscopy) counted. In each experiment, two dishes per parasite culture were prepared, each dish including at least three spots coated with CD36. Ten microscope fields were counted per spot.

### Measurement of Platelet Surface Receptor Proteins CD41, CD62P and CD36 by Flow Cytometry

PRP was prepared from freshly drawn whole blood collected in CPD anticoagulant (“fresh platelets”) or from whole blood from the same donor stored at 4°C for one week (“stored platelets”). The PRP was diluted in modified Tyrode’s buffer (134 mM NaCl; 2.9 mM KCl; 0.34 mM Na_2_HPO_4_^.^12H_2_O; 12 mM NaHCO_3_; HEPES 20 mM; MgCl_2_ 1 mM; glucose 5 mM) at pH 6.8 or pH 7.3 to obtain a final concentration of 1×10^7^ platelets/ml. Diluted platelets at the corresponding pH were incubated for 40 min at room temperature before stimulation with 50 µM TRAP (Thrombin Receptor Activator Peptide, sequence SFLLRN, also known as the PAP1 peptide [15]). FITC-conjugated primary antibodies for CD41 (Mouse Anti-Human CD41-FITC, Clone 5B12, DAKO) and CD62P (Mouse Anti-Human CD62P-FITC, Clone AK-4, BD Pharmingen) were added to the samples and incubated at room temperature for 20 min. Isotype-matched negative controls directly conjugated to FITC were used to assess nonspecific fluorescence. For CD36 detection, primary antibody (Mouse anti-human CD36, clone FA6-152, Immunotech, or an isotype-matched control antibody) was added and incubated for 20 min. Then PE-conjugated secondary antibody was added (polyclonal Rabbit F(ab′)2 anti-mouse Immunoglobulins RPE, DAKO) and incubated for 20 min at room temperature. All samples were fixed in formaldehyde (0.2% final concentration) prior to analysis by BD Biosciences FACSCalibur flow cytometer. The PRP sample at pH 7.3 obtained from fresh whole blood was used to prepare an FSC/SSC density plot, and the platelet population was gated to exclude debris. The same gate was used for the fluorescence analysis of all the samples. A total of 10,000 gated events were counted per experimental condition.

### Graphing and Statistical Analysis

Graphs were prepared and statistical analysis carried out using GraphPad Prism v5 (GraphPad Software Inc, La Jolla, CA).

## Results

### Development of a Selection Method for the Platelet-mediated Clumping Phenotype

A method was developed to separate parasites with the ability to form platelet-mediated clumps (Clump+) from isogenic parasites that do not form platelet-mediated clumps (Clump−). The method was based on magnetic separation using beads coated with an anti-platelet mAb. Once a clumping assay had been performed and aggregates of IEs and platelets were present in the culture (Figure 1A), antibody-coated magnetic beads were added to the suspension, and they bound to clumps through direct recognition of platelets (Figure 1B). A magnet was used to separate the positive fraction containing the clumping IEs, platelets and beads (Figure 1C, bound to the magnet) from the negative fraction (the supernatant) containing the non-clumping IEs and uninfected Es. Repeated rounds of positive or negative selection as described in the methods gave enriched isogenic Clump+ or Clump− parasite lines.

Four different *P. falciparum* laboratory strains (IT, 3D7, HB3, and Dd2) were successfully selected to give positive and negative clumping populations (Table 1). The four lines differed markedly in their starting clumping levels, with the clumping frequency (CF, percentage of IEs forming clumps) of the unselected parasites ranging from 0% in Dd2 to 45% in HB3. However, all strains could be selected to give Clump+ populations with ≥50% CF and Clump− populations with CF<5% (Table 1). IT and 3D7 strains gave the clearest distinction between Clump+ populations (CF 65–85%) and Clump− populations (CF ≤2%), and the CF of the selected populations showed little change for 7–10 days after selection. High CF (65–85%) could also be achieved in parasite strain HB3, however, maintaining a low CF (<5%) was difficult in this strain, as the CF of the Clump− population tended to double within the first 7 days after selection. Dd2 is a parasite strain lacking knobs [16], the surface protrusions of *P. falciparum* IEs where parasite adhesins such as PfEMP1 (encoded by *var* genes) are concentrated [17]. Loss of knobs can occur upon continuous culture of *P. falciparum* [18], [19] due to a deletion of the *KAHRP* gene at the end of chromosome 2 [20]. Knobs are important for adhesion of *P. falciparum* infected erythrocytes under physiologic flow conditions, probably by increasing the avidity of receptor-ligand interactions [21]. The lack of knobs in Dd2 may explain the absence of clumping in unselected Dd2 (Table 1). However, it was possible to select a Dd2 Clump+ population with 50% CF after five rounds of positive selection, showing that knobs are not essential for clumping. For all four strains, clumping in the Clump+ population was dependent upon the presence of platelets, as clumping only occurred in the presence of PRP but not in PPP.

**Table 1:** Summary of clumping selection for four *P. falciparum* strains.

Parasite strain	Unselected	Clump+ population		Clump− population	
	CF^a^ (%)	Rounds of positive selection^b^	CF^a^ (%)	Rounds of negative selection^b^	CF^a^ (%)
IT	20–40	1–2	75–85	2	1–2
HB3	35–45	2–4	65–75	2–3	1.5–8
3D7	5–15	2–3	65–85	1	0–1.5
Dd2	0	5	50	n.a.	n.a.

a CF: Clumping frequency is the percentage of infected erythrocytes involved in clumps of three or more adherent cells.

b The number of rounds of selection that was needed to achieve the desired clumping phenotype is indicated.

n.a. not applicable.

### Knob Positivity of Selected Parasites

**Figure 2:**
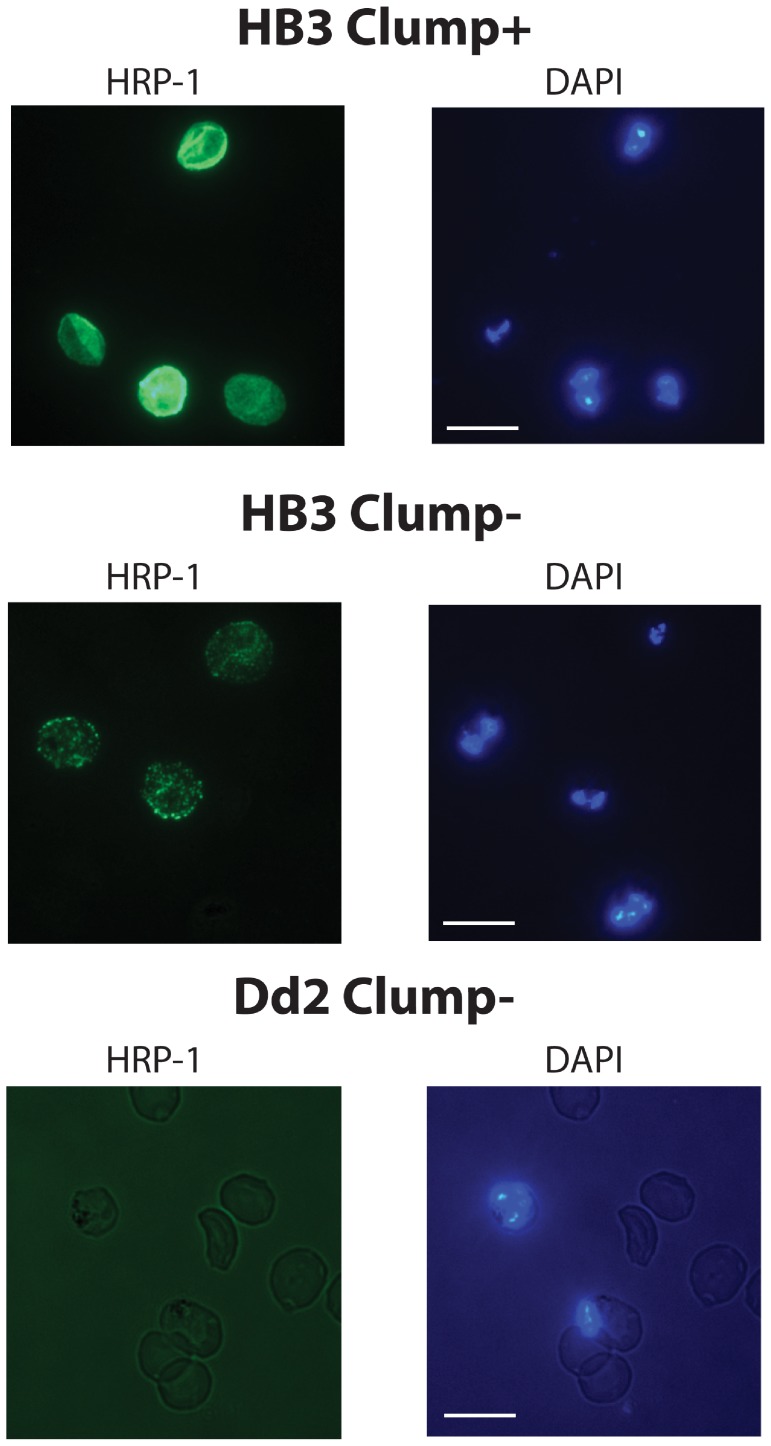
Immunofluorescence assay to detects knobs. Fixed thin blood smears of Clump+ and Clump− parasites were stained with mAb89 to KAHRP (10 µg/ml in PBS/1%BSA) followed by 1/500 dilution of Alexa Fluor 488 highly cross-absorbed goat anti-mouse IgG in PBS/1% BSA (green, left column) plus 1 µg/ml 4,6-DiAmidino-2-PhenylIndole (DAPI) to stain parasite nuclei (blue, right column). No green fluorescence was seen with the knob-negative strain Dd2 (bottom left) nor with the IgG2a isotype control mAb (not shown). In three separate IFAs on parasite cultures from separate days, HB3 Clump+ parasites consistently showed markedly brighter staining (top left) than HB3 Clump− parasites (middle left). Representative images are shown. Scale bar = 10 µM.

Because knobs are known to have a pronounced effect on *P. falciparum* adhesion [21], we tested the Clump+ and Clump− populations of all strains to determine the percentage of knob positive IEs in each case. This was done by Immuno-Fluorescence Assay (IFA) of fixed thin smears using monoclonal antibody (mAb) 89 to the Knob-Associated Histidine Rich Protein (KAHRP) [13]. We found that Dd2 Clump+ and Clump− populations showed 0% knob-positive IEs as expected (Table 2). For the IT strain, Clump+ and Clump− populations did not differ significantly, each having >90% knob-positive IEs (Table 2). For 3D7 and HB3, the proportion of knob-positive IEs in the Clump− population was significantly lower than in the Clump+ population, although in each case, >80% of IEs were knob-positive (Table 2), suggesting that absolute lack of knobs is unlikely to be responsible for the Clump− phenotype. For strain HB3, there was a marked difference in the intensity and pattern of fluorescence in the IFA, with Clump+ parasites showing a bright, smooth, slightly granular appearance, whereas the fluorescence in Clump− parasites was fainter and more punctate (Figure 2). Consistent differences in fluorescence intensity or pattern were not seen for IT and 3D7 parasites, in which both Clump+ and Clump− populations showed a similar appearance to that seen in HB3 Clump+ parasites.

**Table 2:** Percentage of knob-positive infected erythrocytes in *P. falciparum* strains selected for clumping.

Parasite strain	Clump+ population(% KAHRP+)^a^	Clump− population(% KAHRP+)^a^	P value^b^
Dd2	0 (0)	0 (0)	NA^c^
IT	93 (2.2)	97 (0.9)	0.09
3D7	93 (1.8)	81 (2.0)	0.0047
HB3	100 (0.5)	81 (1.8)	<0.0001

a The percentage of infected erythrocytes expressing the Knob-Associated Histidine Rich Protein (KAHRP) was assessed by fixed cell IFA with mAb 89 to KAHRP. 100 cells in four separate areas of the slide were assessed for staining, and the mean (standard error) of the percentage of positive cells from the four counts are shown.

b Paired t test.

c NA: not applicable.

### Characterization of the Platelet Receptors Used for Clumping by the Clump+ Selected Parasites

**Figure 3:**
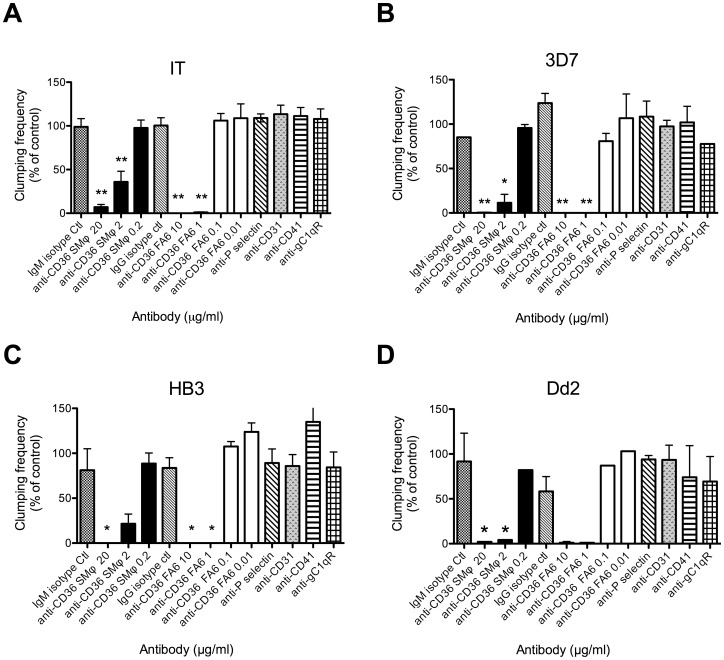
Inhibition of platelet-mediated clumping of *P. falciparum* infected erythrocytes (IEs) by antibodies to platelet receptors. Platelets were pre-incubated with antibodies for 45 mins before being used to set up clumping assays. All antibodies were at 20 µg/ml except anti-CD36 mAb clone SMφ (also 2 and 0.2 µg/ml) and anti-CD36 mAb clone FA6-12 (10, 1, 0.1 and 0.01 µg/ml). Platelet-mediated clumping assays were set up at 10% Ht, 1% Pt, and 20% PRP, at pH7.3, and the clumping frequency (percentage of IEs in clumps out of 500 IEs counted) was assessed by fluorescence microscopy of two wet preparations at time point 1 h. To normalize between experiments with different baseline clumping frequencies, the data are shown as percentage of a control with no added antibody. Data shown are the mean and standard error (SE) of CF. (A) Parasite strain IT (six independent experiments), (B) 3D7 (two experiments), (C) HB3 (three experiments; value of SE for anti-CD41 is 29) and (D) Dd2 (two experiments). One-way ANOVA showed a significant effect of antibodies on *P. falciparum* clumping (p<0.005) for all strains, with CD36 antibodies showing significant inhibition in all cases (Tukey’s multiple comparison test, * p<0.05, ** p<0.005).

The Clump+ selected lines were tested for clumping inhibition with antibodies to platelet receptors to determine the specific clumping phenotype of each strain. Antibodies to the three previously identified platelet surface molecules implicated as receptors for clumping were used (anti-CD36 [1], anti-globular C1q receptor (gC1qR/HABP1/p32) [7], and anti-CD62P (P-selectin) [4]) along with negative control antibodies to platelet surface molecules not implicated in clumping (anti-CD41 (GPIIb) and anti-CD31 (PECAM-1)). Clumping in all four parasite strains was inhibited by two different CD36 antibodies in a dose-dependent manner (mAb clone FA6-12 (IgG1) and mAb clone SMφ (IgM) (Figure 3). Ten µg/ml of mAb FA6-12 was sufficient to completely abolish clumping in all four strains (Figure 3). Antibodies to gC1qR, CD62P, CD41 and CD31 had no significant inhibitory effect on clumping (Figure 3). None of the antibodies caused platelet aggregation at the concentrations used. These data show that the Clump+ selected parasites from IT, 3D7, HB3 and Dd2 are dependent on CD36 for clumping. The lack of effect of antibodies to gC1qR and CD62P, previously shown to inhibit clumping in gC1qR- and CD62P-dependent clumping strains [4], [7], suggests that these receptors are unlikely to play a role in clumping of the Clump+ selected strains derived here.

### Examination of the Effect of pH on Clumping

**Figure 4:**
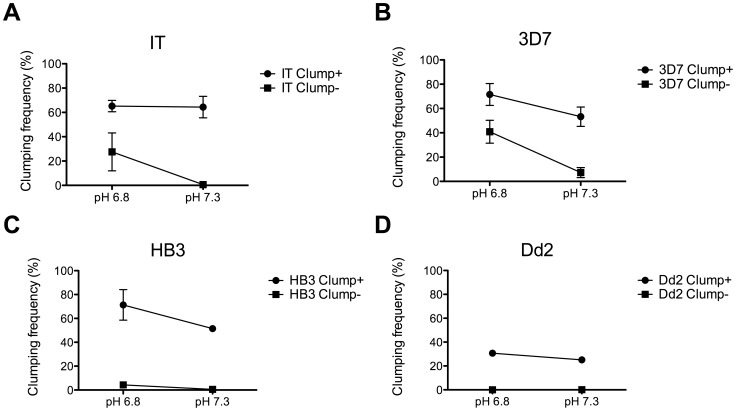
Effect of pH on platelet-mediated clumping of *P. falciparum* infected erythrocytes (IEs). Platelet-mediated clumping assays were set up at 10% Ht, 1% Pt, and 20% PRP, in binding medium at pH 7.3 or pH 6.8. The clumping frequency (CF, percentage of IEs in clumps out of 500 IEs counted) was assessed by fluorescence microscopy of two wet preparations at time point 1 h. Graphs show mean and SE of CF for each parasite strain. (A) IT (4 independent experiments), (B) 3D7 (6 experiments), (C) HB3 (2 experiments) and (D) Dd2 (2 experiments). The CF of 3D7 Clump− parasites showed a significant increase at pH 6.8 compared to 7.3 (p<0.01, paired t test). Other comparisons were not significant.

Adhesion of *P. falciparum* IEs to CD36 measured in standard adhesion assays using recombinant proteins spotted onto plastic or CD36-expressing cell lines is known to be strongly influenced by pH, with significantly higher adhesion occurring at low pH (pH 6.8) than at normal physiological pH (pH 7.3–7.4) [22]. Given that the Clump+ lines selected above all showed CD36-dependent clumping, we investigated whether the pH of the medium would influence the clumping phenotype of the selected parasites (all selection procedures were conducted at a physiological pH of 7.3). For all four strains we compared clumping at pH 7.3 with clumping at pH 6.8. For the Clump+ populations in all cases, we found that lowering the pH had only minor effects on the clumping frequency (Figure 4A–D). The Clump− populations from strains HB3 and Dd2 remained clumping-negative even at low pH (Figure 4C and D), however, for strains IT and 3D7, lowering the pH caused an increase in the clumping frequency of the Clump− populations (Figure 4A and B) which was statistically significant for strain 3D7 (3D7 p<0.01 paired t test, n = 6; IT p = 0.18, paired t test, n = 4). These experiments show that similar to the binding of IEs to CD36-coated surfaces measured in standard adhesion assays, clumping in some strains is influenced by pH, with higher clumping at low pH.

### Characterization of the Ability of Clump+ and Clump− Selected Cultures to Bind to CD36 in Static and Flow Adhesion Assays

**Figure 5:**
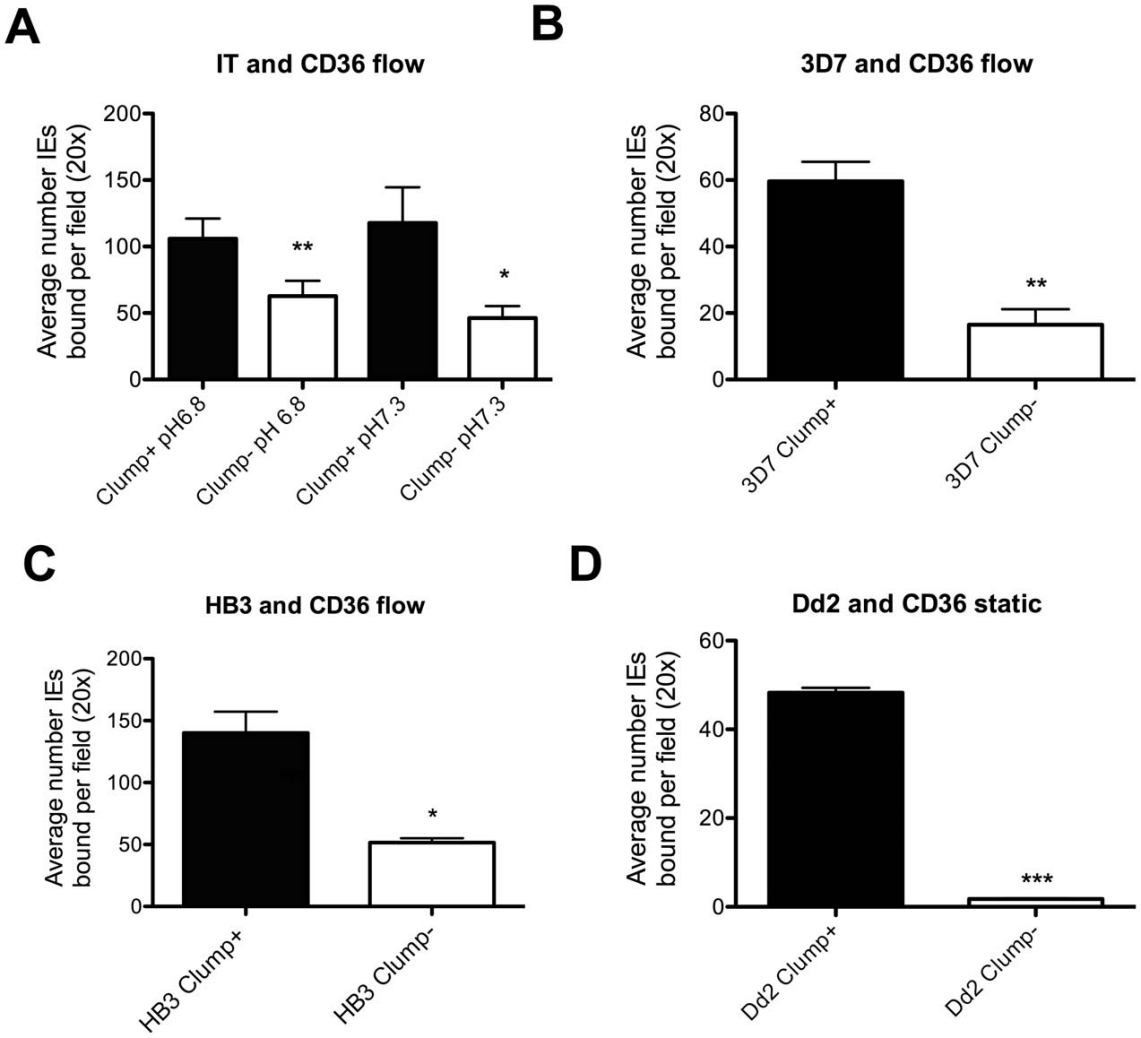
Comparison of CD36-binding by *P. falciparum* Clump+ and Clump − **parasite strains.** (A) Binding of parasite strain IT to CD36 under flow conditions (0.05 Pa). Parasite cultures at 3% Pt, 1% Ht in BM/1%BSA at either pH 7.3 or pH 6.8 were flowed over microslides coated with 5 µg/ml recombinant CD36. After washing away unbound cells, the numbers of stationary cells per 20× magnification microscope field were counted by direct microscopic observation in six separate areas on the microslide. Binding of an uninfected E control suspension to CD36 was negligible in all experiments. Data shown are mean and SE of data from eight experiments. The Clump+ parasites had 56–89% CF and the Clump− parasites had 0–11% CF. Clump+ parasites showed significantly higher binding to CD36 than Clump− parasites at both pH conditions (pH 7.3 p = 0.0247; pH 6.8, p = 0.0008; paired t test, n = 8 at each pH). (B) Binding of parasite strain 3D7 to CD36 under flow conditions (0.05 Pa). Parasite cultures at 3% Pt, 1% Ht in BM/1%BSA at pH 7.3 were flowed over microslides coated with 5 µg/ml recombinant CD36. Washing and counting were as described above. Data shown are mean and SE from six experiments. The Clump+ parasites had 22–39% CF and the Clump− parasites had 0% CF in all experiments. Clump+ parasites showed significantly higher binding than Clump− parasites (p = 0.0032, paired t test) (C) Binding of parasite strain HB3 to CD36 under flow conditions (0.05 Pa). Conditions as in (B). Data shown are mean and SE from four experiments. The Clump+ parasites had 43–62% CF and the Clump− parasites had 0–5% CF. The Clump+ parasites showed significantly higher binding than Clump− parasites (p = 0.0118, paired t test). D) Adhesion of Dd2 IEs to CD36 (25 µg/ml) under static conditions. Binding assays were performed in BM/1% BSA at 2% Ht, 3% Pt, pH 7.0 for 1 h at 37°C. Unbound cells were removed by gentle washing and the bound cells fixed with glutaraldehyde and stained with Giemsa. At least three CD36 spots were counted on at least two dishes for each parasite culture in each experiment, with 10 fields at 1000× magnification (light microscopy) being counted per spot. No adhesion of IE or E to PBS control spots was seen. Clump+ Dd2 parasites (CF 45%) showed significantly higher adhesion to CD36 than Clump− Dd2 parasites (CF 0%) (p<0.0001, paired t test. One representative experiment out of two is shown).

We examined further the relationship between the ability of IEs to form platelet-mediated clumps and the ability to bind to CD36 as determined by standard adhesion assays. We specifically wanted to investigate whether there was a direct and simple relationship between clumping and CD36-binding; in other words, do Clump+ parasites bind CD36 whereas Clump− parasites do not? We found that for strain IT, adhesion of Clump+ parasites to CD36 was significantly higher than adhesion of Clump− parasites at both physiological and low pH (Figure 5A), (pH 7.3 p = 0.0247; pH 6.8, p = 0.0008; paired t test, n = 8 at each pH). However, it is clear from Figure 5A that Clump− parasites are able to bind CD36, albeit at a lower level than Clump+ parasites. Binding of Clump− parasites to CD36 was seen even when the CF of the culture was 0% (two experiments), showing that the relationship between clumping and CD36 binding is not absolute. For strains 3D7 and HB3 a similar relationship was seen, with Clump+ parasites showing significantly higher levels of binding to CD36 than Clump− parasites (Figure 5B, 3D7 p = 0.0032, paired t test, n = 6; Figure 5C, HB3 p = 0.0118, paired t test, n = 4. Both strains tested at pH 7.3 only). The knobless parasite Dd2 showed no adhesion to CD36 of Clump+ or Clump− parasites under flow conditions, therefore we examined adhesion of this parasite in static adhesion assays. We found that Dd2 Clump+ parasites were able to bind to CD36 under static conditions, whereas Dd2 Clump− parasites showed minimal adhesion to CD36 (Figure 5D, p<0.0001, paired t test. One representative experiment out of two is shown).

### Characterization of Surface Marker Expression on Platelet Preparations Used for Parasite Clumping Selection

**Figure 6:**
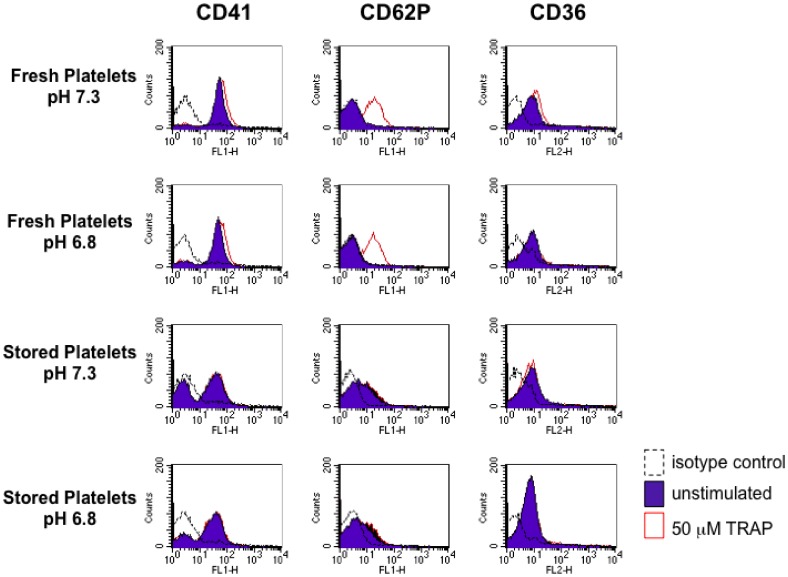
Platelet surface marker expression on fresh and stored platelet preparations. Platelet-rich plasma prepared from freshly drawn whole blood (“fresh platelets”) or from whole blood from the same donor stored at 4°C for one week (“stored platelets”) was diluted in modified Tyrode’s buffer at pH 7.3 or pH 6.8 and incubated for 40 min at room temperature. Surface expression of CD41 (platelet-specific constitutive marker), CD62P (platelet activation marker), and CD36 was assessed by flow cytometry under resting conditions and on platelets stimulated with 50 µM Thrombin Receptor Activator Peptide (TRAP). Specific monoclonal antibodies (mAbs) for each receptor were used along with an isotype control mAb. Data obtained from one donor are shown, and a second independent donor showed very similar results.

Many of the previous studies on platelet-mediated clumping of IEs have been conducted using platelet preparations stored at 4°C (eg. [1], [4], [6]), giving conditions that would be expected to lead to platelet activation [23]. In the selection method developed here, we followed these previous studies and used platelets stored at 4°C. However, concerns about the physiological relevance of stored platelets led us to characterize the expression of potential clumping receptors in stored platelet preparations compared to fresh platelets. We used flow cytometry to examine surface expression of the constitutive platelet surface marker CD41, as well as the clumping receptor and activation marker CD62P and the clumping receptor CD36. In each case, we compared surface expression at pH 7.3 and pH 6.8. Previous work has shown that the clumping receptor gC1qR is expressed at equivalent levels on resting and activated platelets [7], therefore this marker was not examined further. CD41 and CD36 were both detected in fresh and stored platelets, and similar expression levels were seen at pH 7.3 and pH 6.8 (Figure 6). A subpopulation of CD41-negative cells was detected in stored platelet preparations. Expression of the activation marker CD62P on fresh platelets was only detected after platelet activation (using the agonist TRAP peptide, which acts via the platelet thrombin receptor PAR1 [15]). In contrast, CD62P expression was detected on unstimulated stored platelets, and expression levels did not change after TRAP stimulation (Figure 6), suggesting that stored platelets had been activated during storage and remained unresponsive to further stimulation. We also tested whether the clumping phenotype was affected by the use of fresh rather than stored platelets, by investigating whether the CD36 mAb FA6-12 effectively inhibited clumping in Clump+ IT and HB3 parasite lines when fresh platelets were used in the clumping assay. We found that in each case, clumping was completely abolished by 10 and 1 µg/ml of anti-CD36 mAb, giving the same result with fresh platelets as that seen with stored platelets (Figure 3).

## Discussion

A method for positive and negative selection of *P. falciparum* clumping parasites has been developed and applied to show that it is possible to select isogenic Clump+ and Clump− populations from culture-adapted parasite strains. The method makes use of magnetic dynabeads coated with an anti-platelet mAb to select for IEs involved in platelet-mediated clumps. This method is distinct from the MACS-purification method used to select mature IEs on the basis of the iron-containing haemozoin [24], that uses a much stronger magnet to select all pigment-containing IEs. The dynabead method described here gives specific Clump+ and Clump− populations and was used successfully on four commonly used *P. falciparum* laboratory strains (IT, 3D7, HB3 and Dd2).

One concern with the method developed here, and much of the previous published work on *P. falciparum* clumping, is that it has been common practice to use platelets that have been stored at 4°C. This is sub-optimal and non-physiological, because platelets are easily activated, and storage affects their physiology and surface protein expression [23]. We therefore examined the expression of known platelet clumping receptors on both fresh and stored platelets. We found that the clumping receptor CD36 was present and gave similar staining intensity on both fresh and stored platelets (Figure 6), and similar findings have been reported previously for another clumping receptor, gC1qR [7]. However, the CD62P receptor, which is a known platelet activation marker, was not present on fresh platelets unless they were first stimulated with an activator peptide (Figure 6). CD62P was detected on stored platelets, and these platelets were unable to undergo further activation. Therefore it is apparent that although the use of stored platelets may be suitable for the selection of CD36-dependent clumping as shown here, it is likely that physiologically important clumping receptors could be missed by our current method, if they are expressed only on fresh (resting) but not on stored or activated platelets. Furthermore, the antibody we used here for the selections (anti-CD62P) would not be suitable for clumping selection using fresh, resting platelets, unless, as some recent work suggests, fresh platelets become activated on interaction with IEs [4], [25]. We therefore suggest that the method described here should be adapted for future studies investigating other potentially novel clumping phenotypes, by using fresh platelets and anti-CD41 antibody for selections. Another uncertainty in relation to parasite-platelet interactions is whether platelets from malaria patients express different surface markers compared to normal resting platelets. Further characterization of platelet markers in malaria patients would be helpful, and selections could be undertaken with fresh platelets derived directly from patients. Taken together, the importance of considering the physiological relevance of the platelet preparations used and the uncertainty regarding platelet surface marker expression in malaria patients *in vivo*, argues for a more careful use of defined, fresh platelet sources in future studies examining *P. falciparum* clumping phenotypes and their role in malaria disease.

Another concern with the platelet-mediated clumping assay is the wide variation in assay results that occurs with minor modification of the conditions used to set up the assay. In the current work, we found that pH has a profound effect on the clumping phenotype shown by some *P. falciparum* strains. We found that lowering the pH from 7.3 to 6.8 turned a Clump− population into a Clump+ population in some cases (eg. Figure 4B). We have shown previously that experimental conditions such as haematocrit (Ht) and parasitaemia (Pt) have a marked effect on the outcome of clumping assays [2]. For example, an HB3 parasite culture at 1% Pt and 2% Ht showed a clumping frequency of <10%, while the same culture at 1% Pt and 10% Ht, had a clumping frequency of >50%. The effect of pH shown here is yet another example of the variability inherent in the clumping phenotype. This is in contrast to other adhesion phenotypes such as rosetting with uninfected Es, in which the adhesion phenotype is consistent within a single cycle and is not affected by experimental variation in Ht, Pt [26] and pH [27] (although variation in rosette frequency can occur from one asexual cycle to the next due to *var* gene switching). The plasticity in the clumping phenotype in relation to assay conditions means that it is crucial to perform clumping assays in a well-standardized way, especially when multiple experiments are to be compared, or when *P. falciparum* isolates from different clinical malaria groups are to be compared. Moreover, it is fundamental that researchers are aware of the variability inherent in clumping data, especially when comparing studies done by different groups using different conditions.

The variation in clumping assay results under different conditions also raises the question of which, if any, assay conditions are of physiological relevance? Should assays be set up using the Pt and Ht found in malaria patients *in vivo* (although these vary from patient to patient over a wide range)? Furthermore, are *in vitro* clumping assays actually indicative of processes that occur *in vivo*, given that platelet-mediated clumps of the type that develop *in vitro* have never been described in the microvasculature of malaria patients in post-mortem studies [28], [29]? These questions are particularly relevant to studies attempting to address the relationship between clumping and malaria severity, which remains a controversial area. In addition, some studies have shown that interactions between IEs and fresh platelets can lead to parasite-killing *in vitro* [30], [31] and *in vivo* [31], further highlighting the complexity of parasite-platelet interactions. In our view, the *in vitro* clumping assay is a useful tool for investigating the molecular basis of interactions between *P. falciparum* IEs and platelets, however, it is important to bear in mind the uncertainties involved in extrapolating findings from the *in vitro* assay into physiologically relevant observations.

Since the platelet-mediated clumping phenotype was firstly observed [1], [10], [32], it has been described in many but not all *P. falciparum* culture-adapted strains and field isolates [1], [2], [4], [5], [6], [10]. It is not yet clear whether the potential for clumping is restricted to some parasite genotypes, or whether all *P. falciparum* parasites can be selected for this phenotype. The ability to select Clump+ parasites from a knobless *P. falciparum* line such as Dd2, which showed 0% clumping frequency in the starting unselected culture (Table 1), supports the idea that clumping can be selected from all *P. falciparum* genotypes. Furthermore, given that CD36-binding plays a major role in clumping (Figures 3 and 5), and that multiple genes encoding CD36-binding parasite ligands are present in the genome of all *P. falciparum* strains [33], [34], [35], it is plausible that clumping is a common and widespread phenotype.

The four Clump+ selected lines selected here were all dependent on CD36, with clumping being abolished in each case by low concentrations of CD36 mAbs (Figure 3). These data reinforce the importance of CD36 as a platelet receptor for clumping, as described in the seminal paper on platelet-mediated clumping of *P. falciparum* [1]. Although a role for other platelet receptors such as gC1qR [7] and CD62P [4] was not found here, this does not exclude the possibility that other platelet receptors (including gC1qR and CD62P) have key roles in clumping in *P. falciparum* clinical isolates. The present study used a selection method based on whole platelets obtained from stored blood to select parasite populations with the ability to form clumps, whereas a previous study used a purified protein (gC1qR immobilized on plastic) to select a parasite population showing high-level gC1qR binding, that was subsequently found to be capable of clumping when platelets were added to the parasite suspension [7]. Which, if any, clumping phenotype plays an important role in malaria pathogenesis remains to be explored, and it will be interesting to examine the clumping phenotypes of clinical isolates to determine whether any particular phenotype is linked to malaria severity. As published previously [1], the CD41/CD61 complex, which is the most abundant receptor on the surface of platelets and has key functions in platelet aggregation as well as in platelet adhesion to some bacteria [36], does not have a role in platelet-mediated clumping of *P. falciparum* IEs, as antibodies to CD41 do not inhibit clumping (Figure 3). We also found no evidence to support a role for CD62P (P-selectin) or CD31 (PECAM-1) in the Clump+ parasite lines selected here (Figure 3).

This study has reinforced the importance of CD36 as a platelet receptor for clumping. However, it has also indicated that the relationship between CD36-binding and platelet-mediated clumping is not straightforward, because non-clumping parasite populations can still bind to CD36 recombinant protein immobilized on plastic in flow or static adhesion assays (Figure 5). The fact that not all CD36-binding parasites form platelet-mediated clumps is in accordance with previous observations [1]. Possible hypotheses to explain why some but not all CD36-binding parasites form clumps are: 1) Clump+ parasites might have higher affinity for CD36 than Clump− parasites, 2) Clumping may require a parasite ligand that binds CD36 plus a second ligand that binds to another platelet molecule and 3) the parasite could have different molecular pathways (i.e. distinct ligands) for binding to CD36 in clumping compared to binding to CD36-coated surfaces. In relation to this, several proteins on the surface of IEs have been found to bind to CD36 to date, including multiple parasite-derived PfEMP1 variants (encoded by *var* genes) [33], [34] and modified erythrocyte surface molecules such as Band 3 [37]. Further experiments are required in order to test the above hypotheses.

The flow adhesion assays, showing higher binding to CD36 under flow by Clump+ than Clump− parasites (Figure 5) are consistent with hypothesis 1 above, that Clump+ parasites may have higher affinity for CD36 than Clump− parasites. However, direct comparison of binding affinities by methods such as Surface Plasmon Resonance cannot be carried out until the parasite CD36-binding ligands from Clump+ and Clump− parasites are identified. Differing affinities for CD36 could be generated by expression of distinct PfEMP1 variants in Clump+ and Clump− parasites, or by differing levels of surface expression of a parasite CD36-binding ligand (due to transport and trafficking differences between Clump+ and Clump− parasites). Differences in knob expression could also affect CD36-binding affinity, although it is clear from our data that knobs are not essential for clumping because the knobless strain Dd2 could be successfully selected to give Clump+ parasites (Table 1) and the other three strains (IT, 3D7 and HB3) all showed >80% knob positive IEs in both Clump+ and Clump− populations (Table 2). Our data do suggest, however, that for strain HB3, there could be an alteration in the number or distribution of knobs in Clump− compared to Clump+ parasites that might influence the adhesion phenotype (Figure 2). Further experiments will be required to investigate this possibility.

The fact that isogenic Clump+ and Clump− populations were easily obtained here in a small number of rounds of selection is suggestive of differential transcription processes regulating the capacity of IEs for platelet-mediated clumping. The slow changes in CF seen after selection are compatible with the involvement of *var* genes encoding PfEMP1 or other variant surface antigens in platelet-mediated clumping (i.e. changes in CF correlating with *var* gene transcriptional switching) [9]. The methodology detailed here provides the means to select isogenic Clump+ and Clump− parasite lines, which can then be linked to transcriptional profiling and microarray studies to identify parasite adhesion ligands, as has been done with rosetting [33], [34] and brain endothelial cell-binding parasites [35]. The selection method described here provides a powerful tool for future investigations on the molecular mechanisms involved in *P. falciparum* clumping.
